# Thrombo-Inflammation and Immunological Response in Ischemic Stroke: Focusing on Platelet-Tregs Interaction

**DOI:** 10.3389/fncel.2022.955385

**Published:** 2022-06-29

**Authors:** Jieqiong Cui, Huayan Li, Zongning Chen, Ting Dong, Xiying He, Yuanyuan Wei, Zhengkun Li, Jinfeng Duan, Ting Cao, Qian Chen, Dongmei Ma, Yang Zhou, Bo Wang, Mingqin Shi, Qin Zhang, Lei Xiong, Dongdong Qin

**Affiliations:** ^1^School of Basic Medical Sciences, Yunnan University of Chinese Medicine, Kunming, China; ^2^The First School of Clinical Medicine, Yunnan University of Chinese Medicine, Kunming, China; ^3^Department of General Medicine, Lijiang People’s Hospital, Lijiang, China; ^4^Department of Laboratory Medicine, The First People’s Hospital of Yunnan Province, Kunming, China; ^5^School of Chinese Medicine, Yunnan University of Chinese Medicine, Kunming, China

**Keywords:** ischemic stroke, platelets, thromboinflammation, regulatory T cells, immunomodulation

## Abstract

Strokes are mainly caused by thromboembolic obstruction of a major cerebral artery. Major clinical manifestations include paralysis hemiplegia, aphasia, memory, and learning disorders. In the case of ischemic stroke (IS), hyperactive platelets contribute to advancing an acute thrombotic event progression. Therefore, the principal goal of treatment is to recanalize the occluded vessel and restore cerebral blood flow by thrombolysis or mechanical thrombectomy. However, antiplatelets or thrombolytic therapy may increase the risk of bleeding. Beyond the involvement in thrombosis, platelets also contribute to the inflammatory process induced by cerebral ischemia. Platelet-mediated thrombosis and inflammation in IS lie primarily in the interaction of platelet receptors with endothelial cells and immune cells, including T-cells, monocytes/macrophages, and neutrophils. Following revascularization, intervention with conventional antiplatelet medicines such as aspirin or clopidogrel does not substantially diminish infarct development, most likely due to the limited effects on the thrombo-inflammation process. Emerging evidence has shown that T cells, especially regulatory T cells (Tregs), maintain immune homeostasis and suppress immune responses, playing a critical immunomodulatory role in ischemia-reperfusion injury. Hence, considering the deleterious effects of inflammatory and immune responses, there is an urgent need for more targeted agents to limit the thrombotic-inflammatory activity of platelets and minimize the risk of a cerebral hemorrhage. This review highlights the involvement of platelets in neuroinflammation and the evolving role of Tregs and platelets in IS. In response to all issues, preclinical and clinical strategies should generate more viable therapeutics for preventing and managing IS with immunotherapy targeting platelets and Tregs.

## Introduction

According to the Global Burden of Disease Study, the number of deaths from stroke has increased significantly over the last decade, and remained the second-leading cause of death worldwide (GBD 2019 Stroke Collaborators, 2021). Stroke is a heterogeneous disease with acute central nervous system (CNS) focal injury caused by vascular causes, including ischemic stroke (IS), intracerebral hemorrhage (ICH) and pathogenic subtypes (Sacco et al., 2013). The Global Stroke Fact Sheet 2022 states that over 62% of incident strokes are IS (Feigin et al., 2022). Generally, most IS are due to intracranial atherosclerosis and/or *in situ* thrombosis. Furthermore, vascular embolism of atherosclerotic plaques from the aortic arch, carotid artery, or heart also cause IS (Kim et al., 2018; Campbell et al., 2019). Currently, treatments of IS mainly include thrombolysis, antiplatelet, anticoagulation, antithrombosis, and rapid recanalization through mechanical thrombectomy (Crunkhorn, 2018). Mainstay drugs are alteplase, aspirin, clopidogrel hydrogen sulfate, and dual antiplatelet agents (Johnston et al., 2018; Powers, 2020). However, the overall efficacy is limited by the narrow treatment time window, thrombo-inflammation, and ischemia-reperfusion injury. Therefore, it is necessary to combine antiplatelet, anti-inflammatory, and neuroprotective treatments (Stanzione et al., 2020).

**FIGURE 1:**
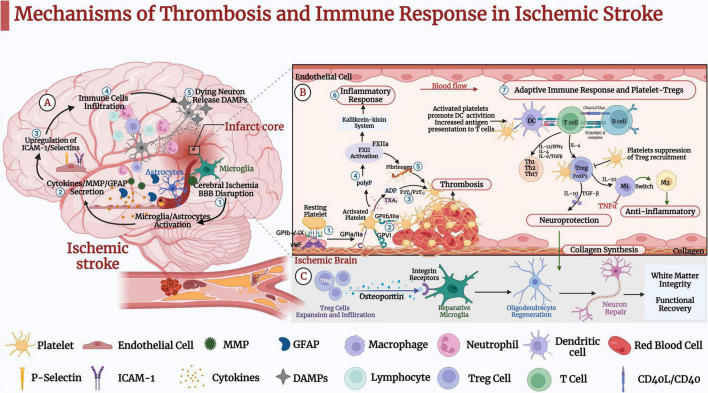
Mechanisms of thrombosis and immune response in ischemic stroke (IS). **(A)** ➀ IS causes oxidative stress and excitotoxicity in the acute phase, activating microglia and astrocytes. ➁ IS leads to the secretion of cytokines, matrix metalloproteinase (MMP), and glial fibrillary acidic protein (GFAP). ➂ Pro-inflammatory factors further lead to upregulation of intercellular cell adhesion molecule (ICAM-1) and selectins on endothelial cells. ➃ Blood-derived inflammatory cells such as neutrophils, macrophages, and lymphocytes infiltrate the ischemic brain tissue. ➄Dead neurons release danger-associated molecular patterns (DAMPs), activating microglia and peripheral immune cells and leading to pro-inflammatory factors that further activate microglia and astrocytes. These pathological events further lead to neuronal death and brain damage. **(B)** Platelet-Tregs Interaction. ➀ At the site of vascular embolic injury, platelets bind to vWF immobilized on collagen *via* the platelet glycoprotein GP Ib-IX-V receptor complex, GPVI and GP la/lla receptors, and bind to exposed collagen to activate adhesion. ➁ Platelets’ activation functionally upregulates GP IIb/IIIa. ➂ Activated platelets release dense granules-ADP, α granules-fibrinogen, vWF, and factor-V, polyphosphates. ➃ Polyphosphates activate coagulation factor XII (FXII). ➄ Activated platelets aggregate *via* fibrinogen and vWF. ➅ In addition to triggering thrombosis through fibrin generation, FXIIa promotes activation of the kallikrein-kinin system and initiates a signaling cascade response, which induces endothelial cell injury, vascular edema, and pro-inflammatory cytokine expression, further induces glial activation, inflammatory response, and ultimately neuronal death. ➆ Activated platelets promote the activation of monocytes and dendritic cells (DCs) and enhance the adaptive immune response. T lymphocytes interact with activated platelets *via* CD40/CD40L to form a solid thrombus. One mechanism by which platelets-derived CD40L can promote atherosclerosis is through inhibiting the migration of Tregs to thrombus sites. Tregs inhibit the expression of adhesion molecules in endothelial and the production of pro-inflammatory cytokines. In addition, it secretes TGF-β and IL-10, promoting collagen synthesis and exerting neuroprotective effects. **(C)** Tregs secrete osteopontin to promote tissue-reparative microglial reactions, thereby promoting oligodendrocyte regeneration as well as remyelination, neuronal repair and behavioral recovery.

In the past decade, molecular and immunological studies have elaborated the additional functions of platelets in the vascular system. Platelets not only play a role in vascular integrity and remodeling, but also they are associated with thrombosis and inflammation, regulating innate or adaptive immunity (Koupenova et al., 2018; Morrell et al., 2019; Hubbard et al., 2021). Platelets support immune responses and maintain immune cell homeostasis to produce and release many immune molecules, directly influencing the development of vascular inflammatory diseases (Morrell et al., 2019). Regulatory T cells are a subset of T cells that control autoimmune responsiveness *in vivo* and are divided into natural regulatory T cells (nTregs) and adaptive regulatory T cells (iTregs). They play a vital role in balancing immune responses by actively suppressing pathogenic immune activation and maintaining immune tolerance (Ferreira et al., 2019). Recent research reports that Tregs infiltrating the brain are critical for behavioral recovery and neural repair (Shi et al., 2021). Therefore, the platelet-immune interface is crucial to understand the pathogenesis of IS and develop optimal treatments. This review briefly summarizes the function of platelet activation and the pathological mechanism of thrombo-inflammation in IS. We also focus on the interaction of platelets with circulating immune cells, especially Tregs, and outline the complex functions of platelets. Furthermore, interventions of IS through targeting platelet-Tregs are also reviewed. In conclusion, the role of platelets in IS and the potential of Tregs as targets for IS intervention require further in-depth investigation (Figure 1). In the future, we hope that more definitive personalized platelets and Tregs-targeted immunotherapy will be able to intervene IS, as well as provide nerve repair and regeneration.

## Platelet Activation and Thrombo-Inflammation in Ischemic Stroke

Platelets are derived from megakaryocyte precursors (Ghoshal and Bhattacharyya, 2014), and δ-granules contain ADP, 5-hydroxytryptamine, polyphosphates, calcium, histamine, and glutamate, which are necessary for hemostasis (Koupenova et al., 2017). Platelet activation processes include intracellular calcium flux, negative translocation of phospholipids, α-granule release, and shape change (Furie and Furie, 2008; Rubenstein and Yin, 2018). The platelet hemostatic program is highly dependent on the glycoprotein receptor complexes GPIb-V-IX and GPVI-FcgR (Borissoff et al., 2011). Recent studies have shown that platelets express more active receptor glycoprotein (GP) VI dimers in stroke patients (Induruwa et al., 2022). Another study combined cell physiology and phosphoproteomics approaches to analyze the downstream signaling mechanism of the immunotyrosine activation motif (ITAM) platelet collagen receptor GPVI. The results inferred more than 300 signaling relationships between effectors (i.e., FcRg, Syk, PLCg2, PKCd, DAPP1) in the platelet GPVI/ITAM response (Babur et al., 2020). Following initial activation, platelets release thromboxane A2 (TxA2) and ADP, which mediate other platelets’ recruitment, activation, and aggregation, culminating in three-dimensional aggregation (Jurk and Kehrel, 2005; Mackman et al., 2007). Thrombin produces fibrin during coagulation or thrombosis, and the fibrinogen receptor GP2b/3a mediates and forms platelet aggregation. However, in a specific pathological condition, the megakaryocyte-platelet-hemostatic axis (MPHA) becomes disturbed, resulting in dysregulated platelet activation and aggregation. This leads to uncontrolled clot formation, cerebrovascular embolism, and the development of IS (Cemin et al., 2011). A recent study found that platelet aggregation is mediated by the endoplasmic reticulum oxidoreductin 1α (Ero1α), protein disulfide isomerase (PDI), and glutathione (GSH) electron transport system on the platelet surface. At the platelet surface, Ero1α constitutively oxidizes PDI and further regulates platelet aggregation in a glutathione-dependent manner (Wang L. et al., 2022). In general, platelet attachment and activation through the GPIb–vWF–GPVI axis and FXII constitute a significant pathological mechanism of acute IS and promote a detrimental inflammatory response.

Reperfusion therapy could assist in restoring blood flow after IS, thereby minimizing brain tissue damage. However, the immune system may still sustain an inflammatory response after recanalization (Alawieh et al., 2020). The interaction of thrombotic and inflammatory mechanisms drives the progression of neuronal damage in IS (Nieswandt et al., 2011). The primary determinant of platelet-mediated inflammation is the platelet adhesion receptors, which can be classified into selectins, integrins, leucine-rich glycoproteins and immunoglobulin-type receptors (Mezger et al., 2015). Atypical platelet function links the cascade of thrombotic reactions to innate immunity through a fundamentally different mechanism. Activated platelets mediate thrombo-inflammation and engage in leukocyte recruitment (Franks et al., 2010). Additionally, other chemicals released by platelets are capable of mediating the kallikrein-kinin pathway, which produces pro-inflammatory bradykinin, causes endothelial cell injury, and leads to cerebrovascular edema and neuronal damage (Dobrivojević et al., 2015; Rawish et al., 2020). Meanwhile, IS activates circulating neutrophils and T cells, leading to a sterile inflammatory response. Platelets coordinate immune cells infiltrating into the brain parenchyma, causing neuronal damage (Konsman et al., 2007). All phases of the IS cascade involve inflammatory signals, from early injury events triggered by cerebral ischemia to the later regenerative processes of neural tissue repair. As the ischemic cascade progresses, neurons rapidly become dysfunctional or die under hypoxic-ischemic conditions, causing activation of the immune system (Lipton, 1999; Kono and Rock, 2008).

## Immunological Response After Ischemic Stroke

After an IS attacks, damaged neurons activate the immune system. While, the over-activated immune system drives the death of neurons. Recent evidence suggests that immune cells, including neutrophils, macrophages, B cells, T cells, and dendritic cells, infiltrate brain tissue after blood-brain barrier disruption (BBB) (Jian et al., 2019). The innate immune system makes the earliest immune response to acute IS *via* pattern recognition of Toll-like receptors (TLRs) and Nod-like receptors (NLRs) (Chamorro et al., 2012). Further, it activates downstream mitogen-activated protein kinase (MAPK) and nuclear factor-kB pathways to produce pro-inflammatory cytokines, reactive oxygen species (ROS), and chemokines, exacerbating post-ischemic inflammation (An et al., 2014). In distinction, the adaptive immune response is relatively delayed in IS. In animal models, neutrophils first migrate to the brain parenchyma after IS, followed by macrophages and natural killer cells, and finally by T and B lymphocyte infiltration. Relevant evidence suggests that adaptive immune responses primarily coordinate T and B lymphocytes and antigen-presenting cells to reduce severe inflammation, and limit the development of ischemic pathology (Gan et al., 2014; Hammond et al., 2014; Fu et al., 2015). A multicenter clinical trial evaluated that the immunomodulator (fingolimod and alteplase) effectively reduced brain injury in patients after acute IS, with suppressed lesion growth from day 1 to day 7 and better recovery at 90 days postoperatively. Patients treated with fingolimod combined with alteplase had lower circulating lymphocytes, smaller lesion volumes (10.1 vs. 34.3 mL; *P* = 0.04), less bleeding (1.2 vs. 4.4 mL; *P* = 0.01), and reduced neurological deficits measured by stroke scale (4 vs. 2; *P* = 0.02) (Zhu et al., 2015). Studies have shown that fingolimod reduces the number of circulating lymphocytes in IS, helps prevent local activation of microglia or macrophages, and controls early infiltration of lymphocytes into brain tissue (Schwab et al., 2005; Massberg and von Andrian, 2006). *In vitro* experiments, the interaction of macrophages with activated platelets enhanced the secretion of pro-inflammatory cytokines, suggesting that activated platelets exacerbate the activation of pro-inflammatory macrophages. In addition, a study found that collagen-activated platelets lead to increased release of interleukin (IL-10) and decreased secretion of tumor necrosis factor alpha (TNF-α) by releasing large amounts of prostaglandin E2 (PGE2) (Linke et al., 2017). In addition to interactions with the innate immune system, platelets interact with dendritic cells (DCs) via the CD40-CD154 axis to promote adaptive immunity (Cognasse et al., 2022). Correlative evidence suggests that immunodeficient Rag1 (–/–), CD4 + T-cell (–/–), and CD8 + T-cell (–/–) mice develop smaller infarct volumes and less leukocyte-platelet adhesion than wild-type mice (Yilmaz et al., 2006). Recent research has shown that mice lacking CD84 (a member of signaling lymphocyte activating molecule) exhibited reduced infiltration of CD4 + T cells, decreased thrombotic activity, and neurological injury after experimental stroke (Schuhmann et al., 2020). Therefore, an in-depth study of the interaction between the platelet-immune system and ischemic brain areas is essential to realize the full potential of immunotherapy in stroke.

## Tregs in Ischemic Stroke

Tregs are an essential subpopulation of T cells that secrete immunosuppressive cytokines and maintain immune homeostasis or limit inflammatory additive injury by inhibiting self-reactive immunity (Sakaguchi, 2000; Duffy et al., 2018). Tregs can be classified into various subtypes such as CD4 + CD25 + Tregs, Tr1, and Th3 cells based on their surface markers, cytokines produced, and mechanism of action. Natural Tregs are usually characterized by CD25 + CD4 + and co-express transcription factor P3 (Foxp3) (Spitz et al., 2016). Currently, FOXP3 + Tregs are the cell line known to maintain immune tolerance, and almost all ongoing clinical trials using cell therapy to induce immune tolerance use CD4 + FOXP3 + Tregs (Ferreira et al., 2019). The inflammatory cascade in IS disrupts the integrity of the BBB. High expression of C-C chemokine receptor 5 (CCR5) on Tregs activates calcium signaling, helping Tregs interfacing with ischemic endothelium and interacting with neutrophils or macrophages (Zhang et al., 2020). Tregs can regulate multiple immune pathways in the pathology of IS through the secretion of cytokines, cytolysis, and receptor pathways. For example, Tregs induce immunosuppression through the production of IL-10 and TGF-β. In addition, Tregs maintain homeostasis of the immune system by decreasing the negative effects of excessive inflammation to prevent brain ischemia and promote neurological repair. Although the exact mechanism by which Tregs regulate IS remains controversial, current relevant studies *in vitro* or *in vivo* suggest that Tregs may be a novel target for IS immunotherapy (Wang H. et al., 2022). In 2009, Liesz et al. (2009b) first investigated the action of Tregs in cerebral ischemia and found that mice lacking Tregs had a significantly larger infarct size than controls at day 7 after permanent middle cerebral artery occlusion. Tregs prevent secondary infarction by inhibiting excessive pro-inflammatory cytokines production and regulating the invasion of lymphocytes or microglia in ischemic brain tissue. In addition, related studies have shown that IL-10 can mediate the protective effects of Tregs after stroke (Planas and Chamorro, 2009). Recent studies have shown that Tregs produce dual regulatory proteins with low affinity for epidermal growth factor receptor (EGFR) ligands to inhibit the proliferation of neurotoxic astrocytes. Furthermore, pathway analysis revealed an enormous enrichment of pathway genes involved in neuroactive ligand-receptor interactions in Tregs (Ito et al., 2019). An animal experiment elucidated the protective effect of IL-2/IL-2Ab (antibody) complexes on the prognosis of IS by expanding the number of Tregs. Results showed that IL-2/IL-2Ab significantly reduced brain infarct volume and suppressed neuroinflammatory responses in mice. Then, the injection of diphtheria toxin (DT) into transgenic mice (DTR mice) to achieve Tregs clearance revealed that the deletion of Tregs eliminated the neuroprotective effect of IL-2/IL-2Ab. Adoptive transfer of Tregs collected from IL-2/IL-2Ab-treated mice showed more effective neuroprotection, suggesting that IL-2/IL-2Ab effectively increased Tregs’ numbers and enhanced function (Zhang et al., 2018).

In addition, a related study also found that increasing Tregs *in vivo via* the JES-6-1/IL-2 complex also reduced neuroinflammatory damage. It confirms that Tregs may promote neurological recovery after IS or prevent recurrent stroke. Li et al. (2013) evaluated the potential and neuroprotective mechanisms of Tregs in an IS model by combined cell-specific deletion, knockout mice, and bone marrow chimeras for Tregs-neutrophils co-culture *in vitro*. Findings suggest that in the early stages of ischemia, adoptive transfer of Tregs reduces infiltration of peripheral inflammatory cells into the injured brain, decreases brain inflammation, and alleviates the impaired BBB integrity. A recent study combining single-cell RNA sequencing and flow cytometry methods found that Tregs began to infiltrate mouse brain tissue 1–5 weeks after experimental stroke. Experimental selective reduction of Tregs impeded oligodendrocyte and white matter repair and functional recovery after stroke. Transcriptome analysis showed that brain-infiltrating Tregs exert potent immunomodulatory effects on monocytes and other immune cells. In addition, Treg-derived bone bridge proteins enhance microglia repair activity via integrin receptors, thereby promoting oligodendrocyte formation and white matter repair (Shi et al., 2021). These results suggest a significant role for Tregs in regulating neuroinflammation, predicting that it may be a promising target for IS.

Tregs have beneficial effects on neural repair after IS, but some studies have shown that they can have the opposite or even no effect (Kleinschnitz et al., 2013). The reason for the inability of Tregs to play an active role may be related to the degree of brain damage (Hug et al., 2009; Liesz et al., 2009a), the timing of Tregs’ analysis (Liesz et al., 2009b; Kleinschnitz et al., 2013; Na et al., 2015; Schuhmann et al., 2015), and inflammatory conditions (Danese and Rutella, 2007). Tregs can play different roles at different stages of IS, and current studies on Tregs migration or proliferation have shown that Tregs have significant anti-IS effects. Therefore, the appropriate timing, dose, and injection site for Tregs expansion need further investigation.

## Platelet-Tregs Interaction Orchestrates Inflammation

As mentioned earlier, platelets are actively involved in immune cell recruitment and host defense in addition to hemostatic function. Related studies have shown that platelets directly influence adaptive immune responses by secreting CD40 and CD40L molecules and partner with a continuous subpopulation of immune cells to coordinate the onset and regression of inflammation (Henn et al., 1998; Lam et al., 2015; Maouia et al., 2020). A previous report found that blocking platelet CD40L may preserve the natural Tregs response, which is a potentially novel mechanism to explain the putative suppressing effects of antiplatelet drugs (Moura and Tjwa, 2010). A study of the kinetic mechanisms by which platelets affect CD4 + T cell responses showed that platelets enhance Tregs responses by promoting the proliferation of FoxP3 + T cells (Zhu et al., 2014). The differentiation of Tregs requires the participation of TGFβ, and a high concentration of TGF-β inhibits IL-23R expression in favor of Foxp3 Tregs (Zhou et al., 2008). Large amounts of TGFβ are stored in platelets and released upon activation at levels approximately 50-fold higher than those released by activated CD4 + T cells. In addition, platelet factor 4 (PF4) significantly promotes Tregs’ differentiation of αCD3/cd28-stimulated CD4 + T cells (Gerdes et al., 2011). One study applied a super-agonist to elucidate the role of Tregs, showing that antibody-mediated Tregs amplification enhanced stroke size and worsened functional outcomes. This suggests that Tregs promote thrombo-inflammatory lesion growth in the acute phase of IS (Schuhmann et al., 2015). The potential of immunomodulatory function is well established, including expansion of Tregs to limit early post-stroke inflammatory cytotoxicity. In addition, testing potential therapeutic approaches using different ischemic models may better approximate the clinical heterogeneity of stroke. Multiple stroke models allow for appropriate modeling of systemic immune changes in all aspects of stroke (Veltkamp et al., 2015). A previous study has shown that platelets promote the resolution of lung inflammation by directly recruiting Tregs into the lung and transcriptionally reprogramming alveolar macrophages to an anti-inflammatory phenotype. This study is the first to demonstrate that platelet MHC I mediates direct and critical regulation of CD8 + T cells, thus breaking the traditional notion that CD8 + T cells are primarily regulated by classical antigen-presenting cells, such as dendritic cells and monocytes. In addition, DQ-OVA was firstly used as a tool protein to demonstrate that platelets can actively endocytose and hydrolyze exogenous antigens, further supporting the crucial regulatory role of platelets. With the widespread application of cellular immunotherapy, the immune regulation induced by platelets will provide new ideas for future T-cell therapy (Kapur and Semple, 2021; Rossaint et al., 2021). This study indicates that platelets orchestrate Tregs and send signals to the macrophages. As the inflammation progresses, platelet-Tregs interaction can affect T cell migration which helps inflammation subside and prevents further tissue damage in IS. A recent study on trauma found that GPIIb/IIIa regulates the interaction between CD4 + Tregs and platelets-, fibrinogen-, and PAR4-dependent pathways (Bock et al., 2022). This provides some reference value to investigate the mechanism of platelet-Tregs interaction in IS.

## Conclusion

Most IS have thromboembolism of the cerebral vessels, and a critical cellular mediator leading to thrombosis is platelets (Jackson, 2011). There are various types of antiplatelet drugs in clinical therapeutic applications, acting on different stages of platelet thrombosis process of adhesion and aggregation and inhibiting relevant receptors or enzymes to achieve antiplatelet effects. Nevertheless, some studies have highlighted that targeting the pathological mechanisms and pathways of IS may be invalid with a single classical antiplatelet agent (Kleinschnitz et al., 2007; Kraft et al., 2015). If innate and adaptive immune cells are over-activated after IS, it could lead to secondary damage and impede neural repair in the brain (An et al., 2014). In addition to novel compounds that impede thrombosis, recent studies have been conducted on novel immunomodulators to treat acute IS (Alawieh et al., 2020). After IS, the immune response involves the central immune cells, peripheral immune cells, BBB, vascular endothelial cells, and various inflammatory molecules. In the past decades, several efforts have tried to find new targets through antiplatelet or immunotherapy to treat IS. Early targeted treatment of thrombo-inflammation during recanalization may be a promising approach. However, most current studies on Thrombo-inflammation are limited to the role of platelets in innate immune responses, such as platelets expressing multiple antigen recognition receptors, platelets regulating the activation and differentiation of monocytes, and platelets interacting with neutrophils. In addition, studies related to the regulation of platelets and T cells and the synergistic effect of platelets and Tregs are still lacking. With the development of gene-editing technologies, platelets can be edited *in vitro*, or generated *in vitro* from megakaryocytes according to their nucleus-free nature, showing a broad prospect as a tool cell for immunotherapy of IS.

## Author Contributions

All authors listed have made a substantial, direct, and intellectual contribution to the work, and approved it for publication.

## Conflict of Interest

The authors declare that the research was conducted in the absence of any commercial or financial relationships that could be construed as a potential conflict of interest.

## Publisher’s Note

All claims expressed in this article are solely those of the authors and do not necessarily represent those of their affiliated organizations, or those of the publisher, the editors and the reviewers. Any product that may be evaluated in this article, or claim that may be made by its manufacturer, is not guaranteed or endorsed by the publisher.
